# Supplementary material for: Time-resolved chemically-selective spectroscopic investigation of the redox reaction between hematite and aluminium
Source: Nat Commun. 2025 Aug 7;16:7282. doi: 10.1038/s41467-025-62436-z (PMC12332039; doi:10.1038/s41467-025-62436-z)
Supplement: Supplementary file 1 — Supplementary Information [file 41467_2025_62436_MOESM1_ESM.pdf]

Supplementary Information for Time-resolved chemically-selective spectroscopic investigation of the redox reaction between hematite and aluminium

Ettore Paltanin[†1,2], Jacopo S. Pelli Cresi[†1], Emiliano Principi[‡1], Wonseok Lee[3], Filippo Bencivenga[1], Dario De Angelis[1,4], Laura Foglia[1], David Garzella[1], Gabor Kurdi[1], Michele Manfredda[1], Denys Naumenko[1,5], Alberto Simoncig[1], Scott K. Cushing[3], Riccardo Mincigrucci[‡1], and Claudio Masciovecchio[1]

[1]Elettra-Sincrotrone Trieste S.C.p.A., 34149 Basovizza, Trieste, Italy
[2]Dipartimento di Fisica, Università degli Studi di Trieste, 34127 Trieste, Italy
[3]Division of Chemistry and Chemical Engineering, California Institute of Technology, 91125 Pasadena, California, USA
[4]CNR - Istituto Officina dei Materiali (IOM), AREA Science Park, Basovizza, 34149 Trieste, Italy
[5]Infineon Technologies, 9500 Villach, Carinthia, Austria

1 Data processing and fitting

For the raster scan pump-probe measurements the transmission of the unperturbed sample, I_{pre}, is measured five times before the pump-probe shot, I_{pp}, and then two measures of the crater generated by the pump beam, I_{post}, are performed as a sanity check of the measurement. This procedure is repeated five times for each pump-probe delay and then processed to obtain an averaged value for I_{pre}, I_{pp} and I_{post} and an error for the transmission value of each delay.

The first step of the data analysis process is the measure of the beamline transmission and a calibration of the correlation between the two spectrometers, Presto (upstream) and WEST (downstream). This is a procedure that is performed without the sample for every photon energy employed in the experiment. The signal for both the spectrometers is obtained by reading the output current of the CCD sensor in full vertical binning mode. The signal is obtained by integrating over the spectral region of interest (ROI). To account for the black noise, the integral over the extracted background baseline is subtracted from the total integral of the signal (see Fig. 2). A correlation function between the counts on PRESTO and the counts on WEST is established in order to get the transmission of the beamline in the working configuration without the sample. The function is determined with a third order polynomial fit, to express the counts on WEST as a function of the counts of PRESTO. A binning is performed on the measured FEL shots, dividing the counts on PRESTO in 100 intervals and taking for each interval the average value of the counts on WEST with the associated standard deviation as error. The fit is then calculated over the binned values, taking the reciprocal of squares of the errors as weights for the individual points (see Fig. 2).

The pump-probe shots are filtered with the quantile approach. The set of pump-probe transmission values are used to generate a set of 10 evenly spaced values (i.e. deciles). The values which are above the 9th quantile or below the 1st quantile are discarded. In this way, the shots whose intensity is significantly different from the median are removed. Accordingly, the preshots and the postshots associated to the removed pump-probe shots are discarded as well. For the filtered shots, the transmission values of the preshots, pump-probe and postshots are plotted against the delays to obtain a comprehensive picture of the measurement at the monitored photon energy. The overall trend could be summarized as follows:

[†]Contributed equally.
[‡]Electronic address: emiliano.principi@elettra.eu; riccardo.mincigrucci@elettra.eu; Corresponding authors

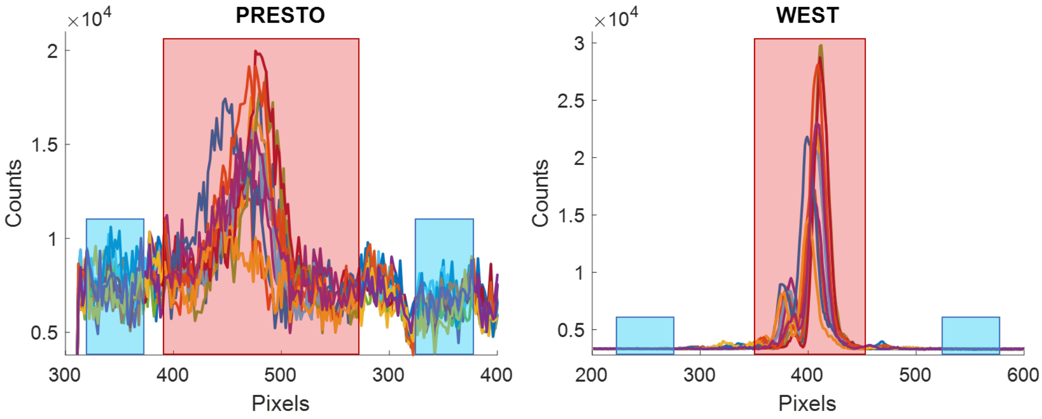

Supplementary Figure 1: Spectra of 10 shots measured with PRESTO (left panel) and with WEST (right panel). The blue rectangles highlight the regions whose area underneath was taken as the background, while the red rectangles highlight the regions whose area underneath was taken to calculate the signal by subtracting the background.

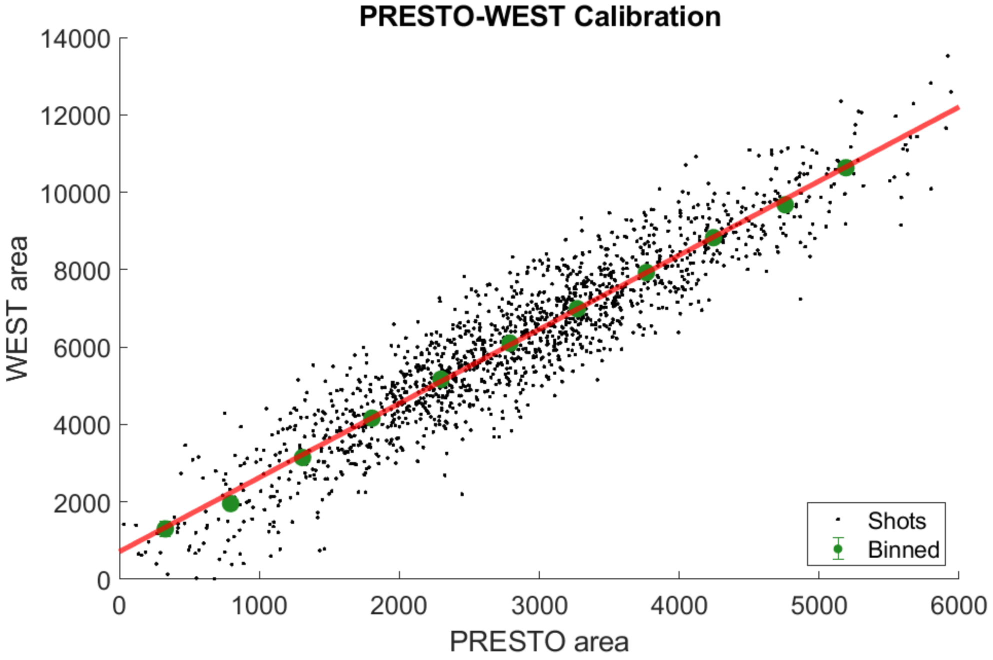

Supplementary Figure 2: Correlation function between the two spectrometers PRESTO and WEST. The black dots are the shots measured, the blue dots are the shots that are filtered out. The red dots with the errorbars are the binned values, while the red line is the fit.

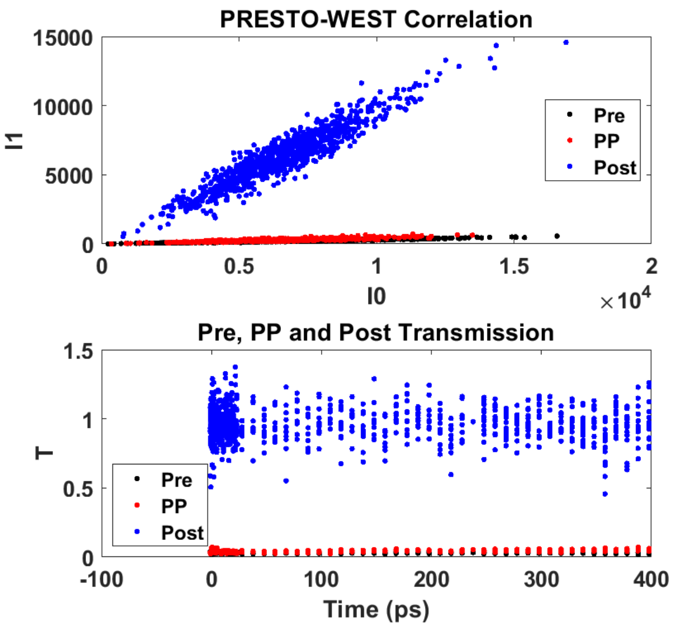

Supplementary Figure 3: Correlation between PRESTO (upstream) and WEST (dowstream) spectrometers (top panel) and unprocessed transmission of the filtered measurements (bottom panel) for preshots, pump-probe and postshots.

- The preshots and the pump-probe shots have an intensity which is significantly lower than the postshots. This is resonable since the preshots and the pump-probe shots interact with the sample, while the postshots basically pass through the experimental chamber unaffected, because the sample is completely ablated by the pump pulse, and hence their transmission is (supposed to be) always close to 1.
- The intensity of the pump-probe shots covers a wider interval with respect to the intensities of the preshots.

The intensity of the WEST spectrometer downstream (I1) has been plotted against the intensity of the PRESTO spectrometer upstream (I0) to check their correlation for the preshots, the pump-probe and the postshots (see Fig. 3).

After the first filtering, a binning procedure has been carried out in order to obtain a single value for the transmission with an associated interval of confidence for each delay where the measurement was performed. The binning is performed individually for preshots, pump-probe and postshots and it consists of taking the average of the transmission values and calculating the error as the standard deviation associated to this collection of values and divide it by the square root of the number of shot minus one, as formalized in the following equation:

$$\mathrm{T_j^{bin}} = \frac{1}{\mathrm{n_j}} \sum_{\mathrm{i=1}}^{\mathrm{n_j}} \mathrm{T_i^j} \qquad\qquad \sigma_{\mathrm{T_j}} = \sqrt{\frac{1}{\mathrm{n_j}-1} \sum_{\mathrm{i=1}}^{\mathrm{n_j}} (\mathrm{T_i^j} - \bar{\mathrm{T}}^{\mathrm{j}})^2} \tag{1}$$

where the index j represents preshots, pump-probe or postshots and n is the number of shots within these groups for a single delay. After binning, we obtained the plots of transmission versus delays that are reported in Fig. 1a.

The normalized variation of transmission of the pump-probe measurements is calculated as follows:

$$\Delta T_{norm} = \frac{\Delta T}{T_{pre}} = \frac{T_{pp} - T_{pre}}{T_{pre}} \tag{2}$$

and the propagation of the errors is calculated accordingly*:

*The variables $\mathrm{T_{pre}}$ and $\mathrm{T_{pp}}$, as well as their standard deviations $\sigma_{\mathrm{T_{pp}}}$ and $\sigma_{\mathrm{T_{pre}}}$, are considered couples of uncorrelated variables. Therefore, the double products are neglected and only the square terms are considered in the error propagation.

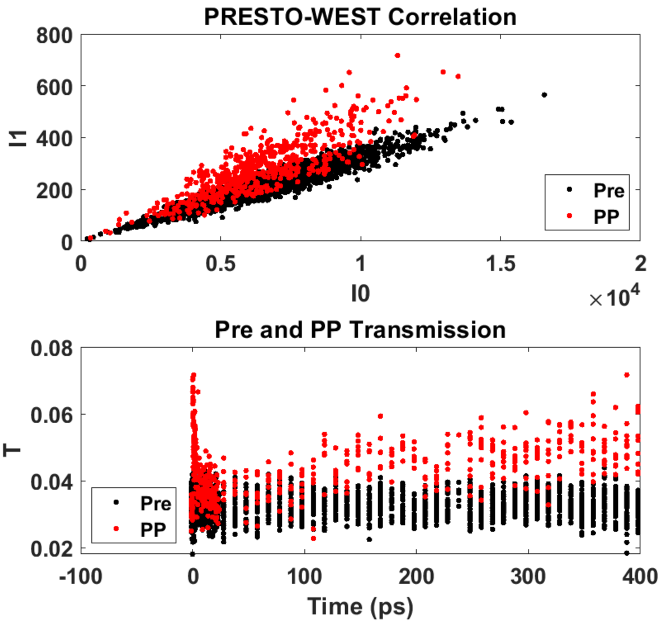

Supplementary Figure 4: Correlation between PRESTO and WEST spectrometers (top panel) and unprocessed transmission of the filtered measurements (bottom panel) preshots and pump-probe.

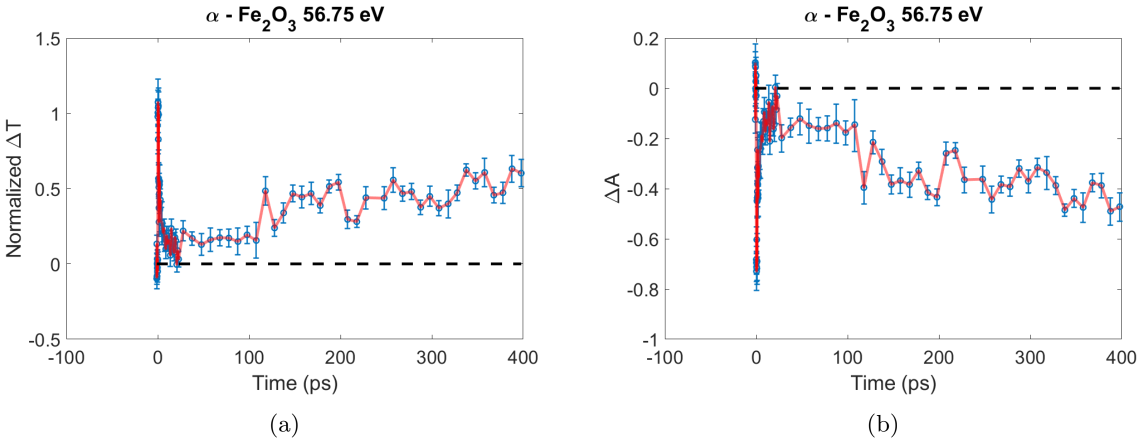

Supplementary Figure 5: (a) Processed transmission and (b) transient absorption for the pump-probe time-dependent traces.

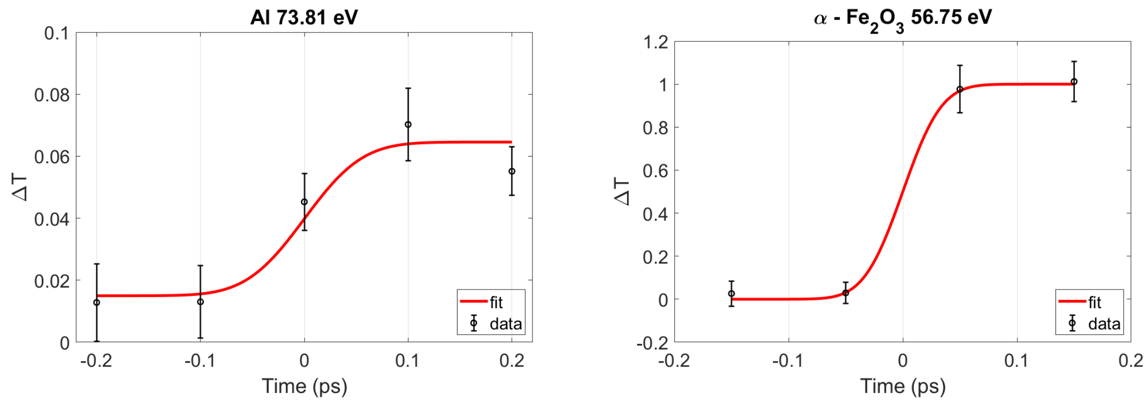

(a) (b)

Supplementary Figure 6: Details of the raise dynamics of (a) Al and (b) α-Fe_2O_3 around delay zero between laser pump and FEL probe. The function used for the fits is described in eq. 7.

$$\begin{aligned}\sigma_{\Delta T} &= \sqrt{\left(\frac{1}{T_{pre}}\cdot\sigma_{T_{pp}}\right)^2+\left(\frac{1}{T_{pre}}\cdot\sigma_{T_{pre}}\right)^2+\frac{2}{T_{pre}}\cdot\sigma_{T_{pre}T_{pp}}+\left[\frac{T_{pp}-T_{pre}}{T_{pre}^2}\cdot\sigma_{T_{pre}}\right]^2}\\ &\approx \sqrt{\left(\frac{1}{T_{pre}}\cdot\sigma_{T_{pp}}\right)^2+\left(\frac{1}{T_{pre}}\cdot\sigma_{T_{pre}}\right)^2+\left(\frac{T_{pp}}{T_{pre}^2}\cdot\sigma_{T_{pre}}\right)^2-\left(\frac{1}{T_{pre}}\cdot\sigma_{T_{pre}}\right)^2}\\ &\approx \sqrt{\left(\frac{1}{T_{pre}}\cdot\sigma_{T_{pp}}\right)^2+\left(\frac{T_{pp}}{T_{pre}^2}\cdot\sigma_{T_{pre}}\right)^2}\end{aligned} \tag{3}$$

The normalized transmission and its associated errors (obtained respectively from Eqs. 2 and 3) are plotted against the pump-probe delays to obtain the refined time-dependent trace reported in Fig. 1a.

The transmission is then manipulated in order to obtain the time-dependent trace in terms of the variation of absorbance (ΔA), calculated as follows:

$$\Delta A = A_{pp} - A_{pre} = \log(1/T_{pp}) - \log(1/T_{pre}) = \log(T_{pre}) - \log(T_{pp}) \tag{4}$$

The interval of confidence of the absorbance values is no longer symmetric with respect to the values themselves because of the logarithm. Hence, the interval of confidence in the absorption scale is computed by taking the logarithm of the upper and lower bounds of the interval of confidence in the transmission scale, as illustrated in the following equation:

$$A_{lower} = -\log(T_{pp} - 0.5\cdot\sigma_{T_{pp}}) \qquad A_{upper} = -\log(T_{pp} + 0.5\cdot\sigma_{T_{pp}}) \tag{5}$$

The absorbance values and their associated intervals of confidence are employed to generate the transient absorption trace reported in Fig. 1b.

Neglecting the jitter between the pump and the probe, which at FERMI is just few femtoseconds, the temporal resolution for the pump-probe signal around t_0 is limited by the convolution between the duration of the pump pulse ($\sigma^t_{pump} \sim 80$ fs) and the probe pulse ($\sigma^t_{probe} \sim 60$ fs):

$$\Delta t_{lim} = \sqrt{[\sigma^t_{pump}]^2 + [\sigma^t_{probe}]^2} = \sqrt{80^2 + 60^2} \simeq 100 \text{ fs} \tag{6}$$

The raise at t0 was fitted for the traces of aluminium and hematite reported in Fig. 6 using an error function:

$$f_r = a\cdot\left[1 + \text{erf}\left(\frac{t}{\sigma\cdot\sqrt{2}}\right)\right] \qquad \text{where} \quad \text{erf}(x) = \frac{1}{\sqrt{2\pi}}\cdot\int_{x_1}^{x_2} e^{-x^2}dx \tag{7}$$

The raise time FWHM was estimated 65 fs for hematite and 102 fs for aluminium.

The reciprocal of the square of the error associated to the normalized transmission of each pump-probe delay were used as weights for the fit of the dynamics of all the measured photon energies. The

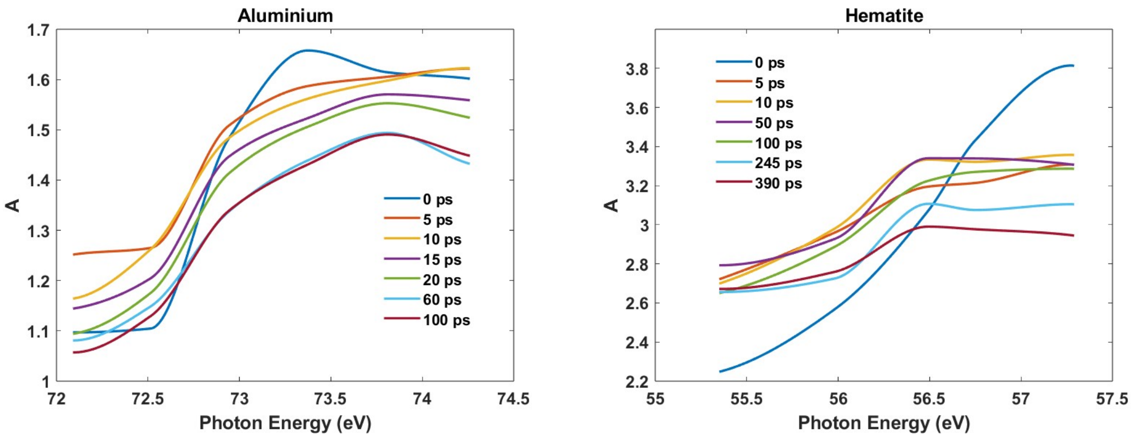

(a) (b)

Supplementary Figure 7: Evolution of the absorption for (a) Al $M_{2,3}$ and (b) Fe $M_{2,3}$ absorption edges at several delays after photothermal excitation with the laser pump at 785 nm. The trend reflects the smearing of the Fermi-Dirac distribution upon the raise of the temperature.

choice for the function used to fit the overall pump probe traces was made with the goal of fitting two processes that occur with different time scales:

- fast dynamic: the equilibration of hot electrons with the lattice via scattering off with optical phonons, which occurs in hundreds of femtoseconds to the first few picoseconds;
- slow dynamic: the dynamics of the lattice which is stressed by the ultrafast raising of the temperature, which occurs in tens to hundreds of picoseconds.

The equation adopted for the fit of the dynamics at energies above the inflection point of the absorption edges was the following:

$$f = \underbrace{\frac{1}{2}\left[1 + \mathrm{erf}\left(\frac{t}{\sigma \cdot \sqrt{2}}\right)\right]}_{\text{time resolution}} \cdot \left[\underbrace{A_1 e^{-\frac{t}{\tau_1}}}_{\text{fast dynamic}} + \underbrace{\frac{A_2}{1 + e^{-\frac{1}{\tau_2}(t-z)}}}_{\text{slow dynamic}}\right] \tag{8}$$

The equation employed to fit the dynamics at energies below the inflection point of the absorption edges was slightly different:

$$f = \underbrace{\frac{1}{2}\left[1 + \mathrm{erf}\left(\frac{t}{\sigma \cdot \sqrt{2}}\right)\right]}_{\text{time resolution}} \cdot \left[\underbrace{\frac{A_1}{1 + e^{-\frac{t}{\tau_1}}}}_{\text{fast dynamic}} + \underbrace{\frac{A_2}{1 + e^{-\frac{1}{\tau_2}(t-z)}}}_{\text{slow dynamic}}\right] \tag{9}$$

Interestingly, for the dynamics measured at 56.49 eV, a different function was used to fit the first decay after t_0, featuring an additional exponential decay term for the fast dynamic (indicated in the following equation with the apex $*$):

$$f = \theta(t) \cdot \underbrace{\frac{1}{2}\left[1 + \mathrm{erf}\left(\frac{t}{\sigma \cdot \sqrt{2}}\right)\right]}_{\text{time resolution}} \cdot \left[\underbrace{A_1 e^{-\frac{t}{\tau_1}} + A_1^* e^{-\frac{t}{\tau_1^*}}}_{\text{fast dynamic}} + \underbrace{\frac{A_2}{1 + e^{-\frac{1}{\tau_2}(t-z)}}}_{\text{slow dynamic}}\right] \tag{10}$$

In Eqs. 8 and 9, t is the independent variable which correspond to the delay between pump and probe beams, σ is the parameter associated to the time resolution that appeared in Eq. 7, A_1 and A_2 are the pre-exponential factors, τ_1 and τ_2 are the kinetic constants for the fast and slow dynamics respectively and z is a parameter that represent the time that the slow lattice dynamic takes to become the dominant contribution. The only difference between the two equations 8 and 9 concerns the term to describe the fast dynamic. In the first case an exponential was chosen in agreement with other transient

absorption studies reported in the literature, but in the second case (i.d. energies below the absorption edge) we opted for a logistic function to perform the fit. The fact that an exponential function with a negative pre-exponential did not work for fitting the dynamics at energies below the absorption edge is probably due to the fact that the trend of the transmission is discordant between the pump-probe traces above and below the absorption edges in the case of the fast dynamic, while it is concordant in the case of the slow dynamic. Anyhow, the fits led to similar kinetic constant regardless the choice of different functional form.

The fitting of the transient absorption spectra was performed using a parametrized sigmoid function (s(x)), which provided the flexibility to adapt to the different shapes of the spectra over the delays investigated. The function adopted is the following:

$$s(x; a_0, b_0, x_0, y_0) = \frac{a_0}{1 + e^{-b_0(x - x_0)}} + y_0 \tag{11}$$

where a_0, b_0, x_0, y_0 are the parameters. This function has been employed in a non-linear least square fitting procedure in order to fit the transient spectra of the individual delays by finding the optimal set of the parameters of the sigmoid function. The weight of each experimental point in the fit has been set as the reciprocal of the associated error on the absorbance. Being the confidence interval of the absorption asymmetric, the error has been considered as the the total uncertainty.

2 Details on the spectrometers

The PRESTO spectrometer is located along the beam transport upstream with respect to the experimetal chamber. It is composed by a diffraction grating, a YAG screen and CCD camera (Hamamatsu). The diffraction grating separates the different spectral components onto the YAG screen, whose fluorescence is captured by the CCD camera, so to avoid damaging of the sensor. The WEST spectrometer, located downstream with respect to the experimental chamber, features a diffraction grating that disperses different wavelengths directly onto the the sensor of the CCD camera (Andor iKon-M). WEST is equipped with a filter wheel before the CCD in order to attenuate the incoming FEL radiation when needed to avoid saturation or damaging of the camera. WEST and PRESTO spectrometers have a resolving power respectively of $\sim 7000 \sim 25000$, which is adequate for monitoring the FEL peaks profile. The combination of two spectrometers, one upstream (I_{up}) and one downstream (I_{down}), allows for drawing a calibration curve for I_{down} as a function of I_{up} without the sample, providing a measure of the transmission efficiency of the beamline for each photon energy employed in the experiment, which is then used during the data processing to isolate the variation of intensity caused by the sample.

3 Sample thickness

The thickness assessment of the double-layer thermite foils used in our XAS measurements in transmission geometry was performed using the CXRO calculator (https : //henke.lbl.gov/optical_constants/filter2.html). In the calculations, densities of $5.26 g/cm^3$ and $2.70 g/cm^3$ were considered for α-Fe_2O_3 and Al, respectively. Oxidation of the free surfaces of the double foil due to atmospheric oxygen was neglected. Figure 8 shows the theoretical absorption of α-Fe_2O_3 and Al thin foils across the Fe $M_{2,3}$ edge and the Al $L_{2,3}$ edge for two different thickness combinations corresponding to the samples used in our experiments:

- absorption of a double-layer consisting of 45 nm α-Fe_2O_3 and 100 nm Al;
- absorption of a double-layer consisting of 20 nm α-Fe_2O_3 and 50 nm Al

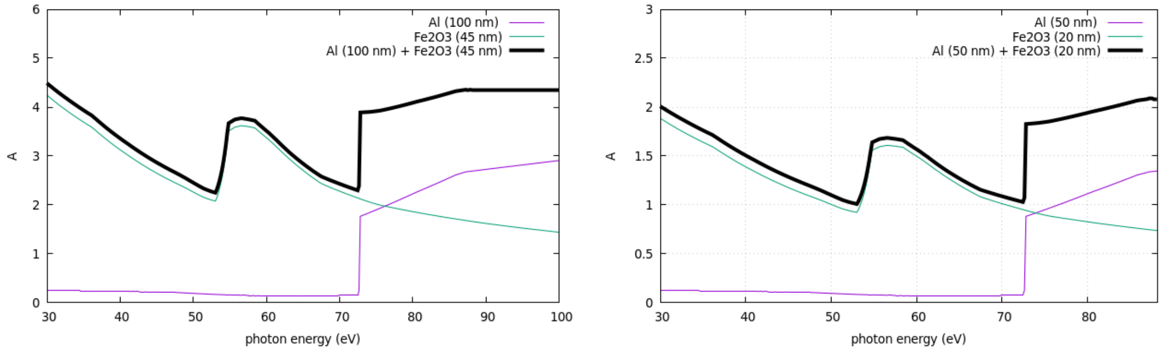

(a)

(b)

Supplementary Figure 8: Theoretical absorption calculation across the Fe $M_{2,3}$ edge and the Al $L_{2,3}$ edge for selected α-Fe_2O_3 and Al nanometric foils.
